# Identification of quantitative trait loci associated with bacterial spot race T4 resistance in intra-specific populations of tomato (*Solanum lycopersicum* L.*)*

**DOI:** 10.1371/journal.pone.0295551

**Published:** 2023-12-11

**Authors:** Pragya Adhikari, Muhammad Irfan Siddique, Frank J. Louws, Dilip R. Panthee

**Affiliations:** 1 Department of Horticultural Science, Mountain Horticultural Crops Research and Extension Center, North Carolina State University, Mills River, North Carolina, United States of America; 2 Bayer Crop Science, Huxley, Iowa, United States of America; 3 Department of Horticultural Science and Department of Entomology and Plant Pathology, North Carolina State University, Raleigh, North Carolina, United States of America; Tocklai Tea Research Institute, INDIA

## Abstract

Bacterial spot of tomato is a serious disease caused by at least four species and four races of *Xanthomonas*- *X*. *euvesicatoria* (race T1), *X*. *vesicatoria* (race T2), *X*. *perforans* (race T3 and T4), and *X*. *gardneri*, with *X*. *perforans* race T4 being predominant in the southeast USA. Practical management of this disease is challenging because of the need for more effective chemicals and commercially resistant cultivars. Identification of genetic resistance is the first step to developing a disease-resistant variety. The objective of this study was to identify quantitative trait loci (QTL) conferring resistance to race T4 in two independent recombinant inbred lines (RILs) populations NC 10204 (intra-specific) and NC 13666 (interspecific) developed by crossing NC 30P x NC22L-1(2008) and NC 1CELBR x PI 270443, respectively. Seven QTLs on chromosomes 2, 6, 7, 11, and 12 were identified in NC 10204. The QTL on chromosome 6 explained the highest percentage of phenotypic variance (up to 21.3%), followed by the QTL on chromosome 12 (up to 8.2%). On the other hand, the QTLs on chromosomes 1, 3, 4, 6, 7, 8, 9, and 11 were detected in NC 13666. The QTLs on chromosomes 6, 7, and 11 were co-located in NC 10204 and NC 13666 populations. The donor of the resistance associated with these QTL in NC 10204 is a released breeding line with superior horticultural traits. Therefore, both the donor parent and the QTL information will be useful in tomato breeding programs as there will be minimal linkage drag associated with the bacterial spot resistance.

## Introduction

Bacterial spot is one of the major foliar diseases of tomatoes in North Carolina (NC), USA, and many other tomato-growing regions worldwide. The disease can potentially cause up to 66% tomato yield loss depending on the growth stage of the infection [1–3]. At least four species within the genus *Xanthomonas*- *X*. *vesicatoria*, *X*. *euvesicatoria*, *X*. *perforans*, and *X*. *gardneri* have been reported as causal agents of bacterial spot [4]. In addition, four races (T1, T2, T3, and T4) associated with differential hosts have been reported [4]. Furthermore, the emergence of race T5 has also been reported in Africa [5]. Practical management of bacterial spots is challenging in commercial production fields due to the limited efficacy of current disease management strategies. Bacterial disease management is mainly based on the integrated use of cultural practices and the application of chemicals such as copper, antibiotics (streptomycin), and plant activators [6]. Nonetheless, *Xanthomonas* isolates resistant to copper and streptomycin have previously been reported in the USA (Florida, Tennessee, Ohio), Brazil, Canada, Ethiopia, and Tanzania [7–11]. We also documented that over 95% of bacterial spot strains are resistant to copper and up to 44% resistant to streptomycin in NC [12].

Although breeding for bacterial spot disease resistance started in 1982, there are no reliable commercial-resistant tomato cultivars available [13]. Breeding for host resistance against a bacterial spot of tomato has been challenging, often impeded by the evolution of new races of the pathogen overcoming the identified resistant germplasm, multi-genic control of the resistance, and a low correlation between seedling assays and field resistance [14]. New races of bacterial spots have evolved and are subsequently discovered during the deployment of commercially resistant cultivars, suggesting the independent evolution of these races in the absence of selection pressure due to host resistance [15, 16]. For example, *X*. *euvesicatoria* was the only species present as race 1 in Florida until 1991 before the *X*. *perforans* race T3 was reported [17]. *X*. *perforans* race T4 strain emerged in 1998 [18, 19] and thereafter has been detected in higher numbers in field surveys in Florida. In a statewide survey across Florida commercial tomato fields, only *X*. *perforans* were identified where races T3 and T4 consisted of 8% and 92% of the population respectively indicating a major shift in the race within *X*. *perforans* [20]. We conducted a systematic survey for the first time in NC and identified single species *X*. *perforans* to be a causal pathogen for bacterial spot disease in tomato fields, within which ~91% belonged to race T4 strains and 9% to race T3 strains [12]. This suggested that the best bacterial spot management practices in tomatoes in NC should be implemented with a major focus on introducing host resistance against race T4.

The hypersensitive response to race T4 has been identified in *S*. *pennellii* LA716 conferred by the locus *RXopJ4/Xv4* [18, 21]. Unfortunately, the *RxopJ4* locus in LA716 is associated with low fruit yield, small fruit, and autogenous leaf necrosis, therefore not suitable for the tomato breeding program [21]. The non-hypersensitive response against race T4 has been detected in PI 114490, HI 7998, PI 126932, and PI 128216. These lines were used to develop three advanced breeding lines 8233, Fla 8517, and Fla 8326 with moderate to a high level of race T4 resistance [14]. However, the T4 resistance in these lines was found to be most dominant with epistatic effect controlled by multiple loci with moderate effects, which limits the development of resistant cultivars from these resistant advanced breeding lines [14].

The complex nature of the disease and the lack of effective bactericides and resistant cultivars necessitate the identification of more resistant tomato genotypes to bacterial spot disease and the study of their underlying genetic architecture. In the present study, we performed QTL (quantitative trait loci) mapping for bacterial spot disease resistance to race T4 in an intra-specific bi-parental fresh market tomato population NC10204 and inter-specific population NC 13666. The NC 10204 was developed crossing parental lines NC 30P and NC 22L-1(2008). Both parental lines showed moderate to partial resistance against bacterial spot disease. Whereas, NC 13666 was developed by a susceptible parent NC 1CELBR and resistant *S*. *pimpinellifolium* accession PI 270443. The main objective of this research was to identify QTL for bacterial spot disease resistance against race T4. The use of elite breeding materials in the QTL mapping study will facilitate the detection of QTL of direct relevance to breeders [22]. In addition, the use of elite breeding materials will allow the detection of the minor allele effects, as most large-effect alleles are homozygous in the population derived from closely related parents [23].

## Materials and methods

### Population development and experimental design

Two mapping populations- NC 10204 and NC 13666 were developed to identify the QTLs associated with the bacterial spot disease race T4 resistance, respectively. The F_1_ hybrid of NC 10204 was created in 2010 by crossing two moderately resistant breeding lines—the plum tomato breeding line NC 30P and the grape tomato breeding line NC 22L-1(2008). NC 30P belongs to cultivated species and has already been released as a breeding line for its superior horticultural traits from the North Carolina State University tomato breeding program [24]. NC 30P when evaluated in 2016 and 2017 replicated trials showed a moderate level of resistance to bacterial spot and has also contributed to BS resistance in commercially released hybrid Plum Regal [24]. NC 22L-1(2008) is an advanced breeding line, which was derived from *S*. *pimpinellifolium* L3707. NC 22L-1(2008) has shown partial resistance to race T4 in the previous field study [25]. The F_1_ hybrid was self-pollinated to create a segregating F_2_ population. The seeds of F_2_ plants were individually extracted to create F_2:3_ families. The single seed descent method was used to create F_5:6_ families at the Mountain Horticultural Crops Research and Extension Center (MHCREC) in Mills River, NC. Although the mapping population started with 284 F_2_ individuals, it was reduced to 121 lines in the F_4:5_ generation and 117 lines in the F_5:6_ generation due to poor seed germination at various generations.

The F_1_ hybrid of NC 13666 was created in 2013 by crossing susceptible tomato breeding line NC 1CELBR and resistant *S*. *pimpinellifolium* accession PI 270443. NC 1CELBR belongs to a cultivated species, which was released as a breeding line for its superior horticultural traits and resistance to early blight and late blight diseases [24]. PI 270443 showed resistance to bacterial spot disease in preliminary disease screening experiments conducted in phytotron and greenhouse [26]. The seed of F_2_ plants was individually extracted to create F_2:3_ families. The single seed descent method was used to create F_6:7_ families of 210 lines at the MHCREC in Mills River, NC.

For the QTL mapping study, we evaluated NC 10204 in four environments and NC 13666 in two environments for BS disease resistance. A total of 121 F_4:5_ and 117 F_5:6_ NC 10204 recombinant inbred lines (RIL) were evaluated during the 2016 and 2017 field seasons, respectively in two locations: MHCREC, Mills River, NC; and Piedmont Research Station (PRS), Salisbury, NC. A total of 210 F_6:7_ RIL of NC 13666 was evaluated in the same two locations in 2017. We also included parental lines, an F_1_ hybrid, and resistant and susceptible checks in each trial. Seeds were germinated in 72 cell trays (56 x 28 cm^2^) in potting mix and grown for six weeks before transplantation in the field. In each trial or environment tested, both populations were planted in a randomized complete block design with two replications with one six-plant plot per line per replication. Individual plants were grown 45 cm apart within rows, and 150 cm apart between rows, in raised beds covered with plastic mulch with drip irrigation. Plants were hand strung and sprayed according to the recommended schedule for fungicides and insecticides [27]. No bacterial disease control measures were taken.

### Inoculum preparation, inoculation, and disease evaluation

The inoculum was produced by culturing a virulent strain of *X*. *perforans* race T4 (#Isolate9) on Yeast extract-dextrose-CaCO3 (YDC) media for 24–36 h at 27°C in an incubator. The isolate9 was collected from one of the growers in Henderson County, NC, and found to be one of the most virulent among several isolates and is being used for inoculation for screening of lines on a regular basis in the tomato breeding program since. The cultured bacteria were washed and suspended in distilled water. The bacterial suspension was standardized to A_600_ = 0.3 (corresponds to a concentration of 5 X 10^8^ CFU/ml). The inoculum was applied at this concentration to susceptible lines NC 84173 and Bonny Best in the greenhouse by misting the foliage until runoff with a backpack sprayer one week before field planting. High humidity was maintained in the greenhouse by misting the foliage with water and covering the plants with plastic for three days before inoculation and 48 hours post-inoculation. Inoculated plants were allowed to develop disease symptoms for a week and then introduced to the fields. Inoculated NC 84173 and Bonny Best were introduced in the field to spread the inoculum in 2016 and 2017 respectively. An inoculated plant of each line was planted at both ends of all individual plots. Plants were rated for disease severity in the field based on the average rating of the plot, using the [28] scale with slight modification, where 0 = 0%, 1 = less than 1%, 2 = 1–3%, 3 = 3–6%, 4 = 6–12%, 5 = 12–25%, 6 = 25–50%, 7 = 50–75%, 8 = 75–87%, 9 = 87–94%, 10 = 94–97%, 11 = 97–100%, and 12 = 100% diseased tissue. The disease was scored five times on a weekly basis starting from 20 days after planting (DAP), and the AUDPC value was calculated as [29]:

$$AUDPC=\sum_{i=1}^{n-1}\left\{\left(\frac{{Score}_{i}+{Score}_{i+1}}{2}\right)*\left({time}_{i+1}-{time}_{i}\right)\right\}$$

Where, *Score*_*i*_ is a disease score at the i^th^ observation, *time*_*i*_ is time in days at the i^th^ observation, and n is the total number of observations.

### Statistical analysis

Data analysis was carried out in SAS version 9.4 [30] and R program (V 4.0.1). The visual illustration of the correlation matrix and principal component analysis was done by using the R language v3.2.3 coupled with the RStudio interface v1.0.143 and R packages ("FactoMineR", "factoextra", "ggplot2", "ggplots", "corrplot"), respectively [31, 32]. The summary statistics and normal probability plots were calculated using the UNIVARIATE procedure of SAS. The heritability was estimated for each environment by calculating variance components using the ‘ASYCOV’ function in PROC MIXED in SAS. Analysis of variance (ANOVA) was performed using the MIXED procedure of SAS for AUDPC data and individual week disease data to determine the differences among genotypes using the following models:

Response=μ+Geno+Year+Location(Year)+Geno*Location(Year)+Error

Where genotype was used as a fixed effect and the rest of the factors were fit as random effects. The least-square means (LSMeans) of each genotype within each environment were calculated using the same model as that of ANOVA and used for QTL analysis. In the case of the NC 13666 population, the model component ‘*Year’* was dropped since it was evaluated for only one year.

### Genotyping, linkage map construction, and QTL analysis

The genomic DNA of NC 10204 (192 lines) and NC 13666 population (232 lines) along with the parental lines were extracted. A modified cetyltrimethyl ammonium bromide method was used for DNA extraction and the DNA samples were stored at -20°C in 10 mM Tris–HCl pH 8.0, and 1 mM EDTA [33]. The entire extracted DNA samples were quantified using a NanoDrop 2000 Spectrophotometer (Thermo Scientific, Wilmington, Delaware, USA). The genomic DNA of 192 lines of NC 10204 along with the parental lines were genotyped using the Solanaceae Coordinated Agricultural Project (SolCAP) 7720 SNP array [34, 35]. SNP genotypes were determined using GenomeStudio version 1.0 (Illumina Inc, San Diego, CA, USA).

A total of 110 individuals from NC 10204 and 210 individuals from NC 13666 were used for the construction of the linkage map because of missing genotypic or phenotypic data of the remaining individuals. Only polymorphic markers were used to construct a final map. The linkage map of NC 10204 was constructed using Joinmap 4.0 [36] and has already been published by [37]. The NC 13666 linkage map was constructed using the R package ‘R/qtl’ [38] to check if we can get a consistent QTL despite different software. The segregation of each marker for the goodness of fit between the observed and the expected Mendelian ratio was tested by χ^2^-test and markers with the distorted segregation at *p <0*.*0001* were discarded. The SNP markers were grouped into their respective chromosome according to tomato genome information SL3.0 available in the Sol Genomics Network (SGN) [39]. The marker orders within each chromosome were calculated using a regression mapping algorithm [40]. Kosambi mapping function was used for the estimation of map distances (cM) [41].

QTL analysis was conducted using Windows QTL Cartographer v 2.5 [42] software. The Composite Interval Mapping (CIM) method was used with the default parameters (model 6). A backward regression was used to perform the CIM analysis to enter or remove background markers from the model. The walking speed was set at one cM for the detection of QTL. A default threshold of the logarithm of odds ratio (LOD) score of 3 was used to declare the presence of QTL [43, 44]. The additive effect and the proportion of the phenotypic variation (R^2^) for each QTL were also obtained using this software. The dominant effect was not reported as individuals used were from the RIL population that was mostly in homozygous condition. QTL explaining more than 10% of the phenotypic variance was considered as major QTL [43, 44]. Considering the bi-parental mapping population and size of the mapping of the population used in this study, any QTL within 10 cM distance on the same chromosomes was regarded as a single QTL, and if detected in at least two environments considered as a consistent QTL [45].

## Results

### Phenotypic variation

The disease data were recorded from four environments for NC 10204 and two environments for NC 13666. The summary statistics of the disease data in NC 10204 and NC 13666, both AUDPC and individual week data for each environment are presented in Tables 1 and 2 respectively. In NC 10204 population, the highest mean AUPDC (201) was observed in PRS at Salisbury, NC in 2016. In both years, disease severity was higher in PRS. The disease severity in MHCREC at Mills River, NC was higher in 2017 compared to 2016, whereas more disease was observed in 2016 in PRS compared to 2017. The disease severity was observed in an increasing trend every week except in PRS in 2017, where disease severity tended to decrease after the third week (Table 1). The genotypic effect was significant for the disease severity data at *p<0*.*05* except for the disease data observed in the first three weeks and AUDPC in MHCREC in 2016, and in the second week in PRS in 2017 (Table 1). Therefore, the disease severity data from the final observations were used for further QTL analysis.

**Table 1:** Descriptive statistics (mean, minimum, maximum, and standard deviation) for bacterial spot disease data of NC 10204, heritability (H^2^), and analysis of variance (ANOVA) for two years (2016 and 2017), and two locations (MHCREC and PRS). AUDPC values were secured based on the summation of 5 weeks of observations. SCORE1-5 represents the [28] severity value for each given week 1 to 5. Y1L1 indicates the MHCREC location and year 2016; Y1L2 indicates the PRS location and year 2016; Y2L1 indicates the MHCREC location and year 2017; Y2L2 indicates the PRS location and year 2017.

Variables	Descriptive Statistics				H^2^	Effect of the fixed variable (ANOVA)					
	Mean	Std. Dev.	Min.	Max.		Source	DF	MS	Error DF	F Value	Pr > F
**AUDPC_Y1L1**	134.7	20.7	81.0	178.0	0.1	Genotype	117.0	456.5	105.0	1.2	0.2012
**AUDPC_Y1L2**	200.8	15.3	169.0	262.0	0.5	Genotype	104.0	349.0	97.0	3.0	< .0001
**AUDPC_Y2L1**	149.0	10.8	112.0	189.0	0.5	Genotype	115.0	178.8	115.0	3.3	< .0001
**AUDPC_Y2L2**	186.5	12.9	147.0	238.0	0.4	Genotype	116.0	224.9	107.0	2.6	< .0001
**SCORE1_Y1L1**	3.3	1.4	1.0	6.0	0.0	Genotype	117.0	2.0	105.0	1.0	0.5367
**SCORE1_Y1L2**	6.8	0.7	4.0	8.0	0.2	Genotype	104.0	0.7	97.0	2.2	< .0001
**SCORE1_Y2L1**	3.9	0.5	2.0	5.0	0.2	Genotype	115.0	0.3	115.0	1.5	0.0188
**SCORE1_Y2L2**	4.7	0.8	2.0	6.0	0.2	Genotype	116.0	0.8	107.0	1.5	0.0227
**SCORE2_Y1L1**	4.0	1.2	1.0	6.0	0.0	Genotype	117.0	1.3	105.0	1.0	0.5587
**SCORE2_Y1L2**	6.8	0.6	4.0	8.0	0.2	Genotype	104.0	0.4	97.0	1.6	0.0102
**SCORE2_Y2L1**	4.6	0.6	3.0	6.0	0.2	Genotype	115.0	0.5	115.0	2.0	0.0002
**SCORE2_Y2L2**	6.1	0.7	4.0	8.0	0.1	Genotype	116.0	0.5	107.0	1.2	0.1798
**SCORE3_Y1L1**	4.8	0.9	2.0	7.0	0.1	Genotype	117.0	0.8	105.0	1.3	0.0918
**SCORE3_Y1L2**	7.0	0.8	6.0	10.0	0.3	Genotype	104.0	0.9	97.0	1.9	0.0008
**SCORE3_Y2L1**	5.0	0.6	3.0	6.0	0.2	Genotype	115.0	0.4	115.0	1.7	0.002
**SCORE3_Y2L2**	7.6	0.8	5.0	10.0	0.3	Genotype	116.0	0.8	107.0	2.1	< .0001
**SCORE4_Y1L1**	5.9	0.8	4.0	7.5	0.2	Genotype	117.0	0.7	105.0	1.4	0.0312
**SCORE4_Y1L2**	7.5	0.9	6.0	11.0	0.5	Genotype	104.0	1.1	97.0	2.9	< .0001
**SCORE4_Y2L1**	6.1	0.9	4.0	9.0	0.0	Genotype	115.0	0.8	115.0	1.7	0.0017
**SCORE4_Y2L2**	7.1	0.6	5.0	10.0	0.5	Genotype	116.0	0.6	107.0	3.1	< .0001
**SCORE5_Y1L1**	6.3	0.7	5.0	8.0	0.3	Genotype	117.0	0.5	105.0	1.8	0.0016
**SCORE5_Y1L2**	7.6	0.9	6.0	11.0	0.5	Genotype	104.0	1.1	97.0	2.9	< .0001
**SCORE5_Y2L1**	7.1	0.5	6.0	9.0	0.4	Genotype	115.0	0.4	115.0	2.3	< .0001
**SCORE5_Y2L2**	7.0	0.5	6.0	9.0	0.2	Genotype	116.0	0.4	107.0	1.6	0.0109

**Table 2:** Descriptive statistics (mean, minimum, maximum, and standard deviation) for bacterial spot disease data of NC 13666, heritability (H^2^), and analysis of variance (ANOVA) for two locations (MHCREC and PRS) in the year 2017. AUDPC values were secured based on the summation of 5 weeks of observations. SCORE1-5 represents the [28] severity value for each given week 1 to 5. L1 indicates the MHREC location; L2 indicates the PRS location.

Variable	Descriptive Statistic				H^2^	Effect of fixed variable (ANOVA)					
	Mean	Std. Dev	Min.	Max.		Source	DF	MS	Error DF	F Value	Pr > F
AUDPCL1	172.3	19.93104	80.5	217.0	0.5	Genotype	208.0	602.9	208.0	3.3	< .0001
AUDPCL2	162.0	14.9	119.0	213.5	0.6	Genotype	206.0	336.9	180.0	3.8	< .0001
SCORE1L1	3.7	0.7	2.0	8.0	0.3	Genotype	208.0	0.6	208.0	1.8	< .0001
SCORE1L2	3.5	0.8	1.0	6.0	0.2	Genotype	206.0	0.8	180.0	1.5	0.0038
SCORE2L1	3.8	0.7	1.0	6.0	0.3	Genotype	208.0	0.7	208.0	2.0	< .0001
SCORE2L2	4.9	0.8	2.0	8.0	0.3	Genotype	206.0	0.8	180.0	2.0	< .0001
SCORE3L1	4.4	0.7	1.0	6.0	0.3	Genotype	208.0	0.7	208.0	1.9	< .0001
SCORE3L2	6.4	0.7	4.0	8.0	0.4	Genotype	206.0	0.7	180.0	2.3	< .0001
SCORE4L1	4.9	0.9	1.0	7.0	0.5	Genotype	208.0	1.1	208.0	3.0	< .0001
SCORE4L2	6.8	0.7	5.0	9.0	0.5	Genotype	206.0	0.8	180.0	3.0	< .0001
SCORE5L1	6.1	0.8	4.0	8.0	0.4	Genotype	208.0	0.8	208.0	2.3	< .0001
SCORE5L2	6.6	0.7	5.0	10.0	0.5	Genotype	206.0	0.6	180.0	2.9	< .0001

**Fig 1:**
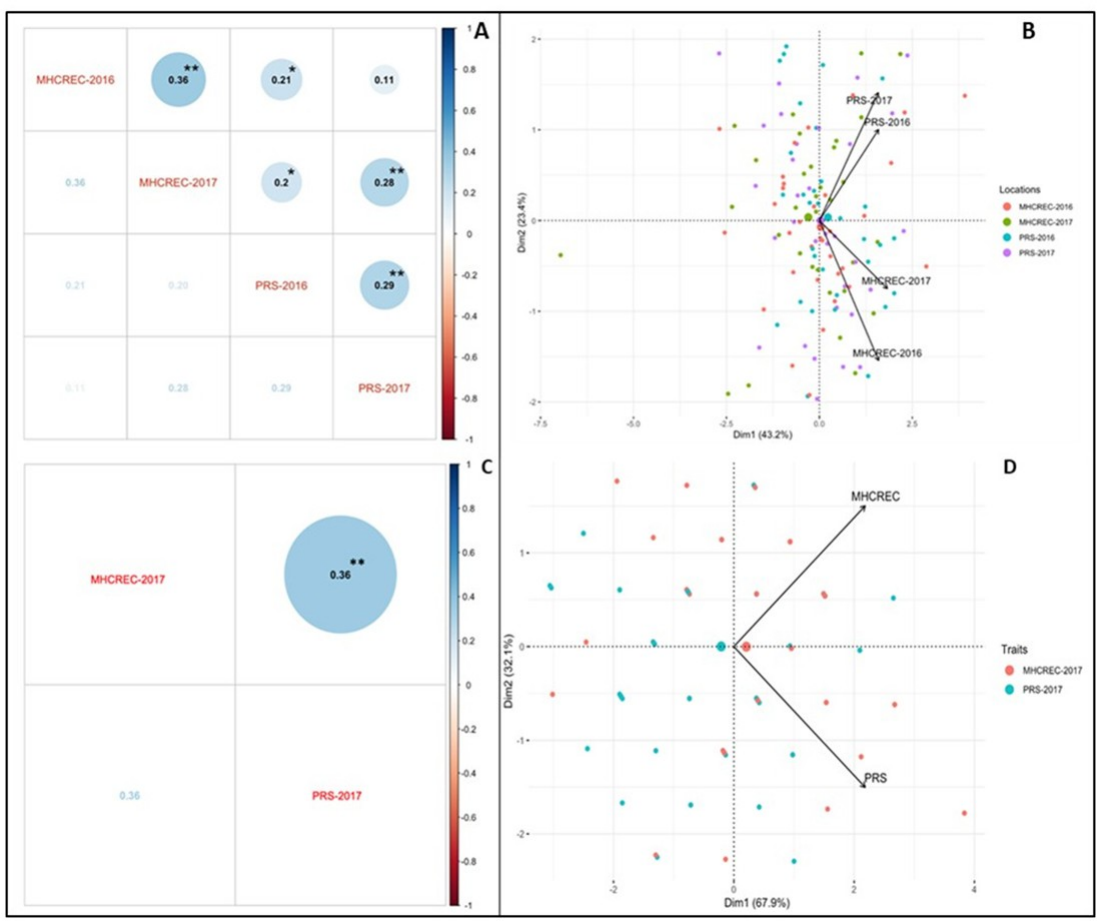
Analysis of phenotypic variability for bacterial spot resistance in mapping population NC10204 and NC 13666. **A)** Pearson’s correlation of AUDPC is significant at both **p < 0.01 and *p < 0.05 levels in NC 10204. **B)** Principal component analysis (PCA) explaining the potential phenotypic variability in NC 10204. **C)** Pearson’s correlation is significant at both **p < 0.01 and *p < 0.05 levels in NC 13666). **D)** Principal component analysis (PCA) explaining the potential phenotypic variability (NC 13666).

The heritability estimates of disease data were low to moderate in NC 10204 population that ranged from 0.1 to 0.5 (Table 1). The AUDPC values were significantly correlated at *p<0*.*05* between years in both locations (MHCREC and PRS), but no correlation was observed between the two locations in separate years (Fig 1A). In both locations, disease severity data scored in the first and second weeks were not significantly correlated between years, but the correlation was observed after the second week except in the fourth week in PRS (Fig 1A and S1 Table). The PCA bi-plot also showed the possible coalition and a total of 66.6% phenotypic variability was observed between the data sets of bacterial spot (BS) resistance in different environments and locations (Fig 1B). The dimension of the first PC (Dim1) broadly outlined and explained 43.2% of the phenotypic variability for BS resistance at MHCREC-2016 and MHCREC-2017 (Fig 1B). The dimension of the second PC (Dim2) also distinguished the 23.4% phenotypic variability for BS resistance (PRS-2016 and PRS-2017) at opposite angles of the PCA bi-plot (Fig 1B).

In NC 13666, the highest AUDPC means (172) was observed in MHCREC (Table 2). There was a significant difference (*p < 0*.*05*) among genotypes of the RIL population of NC 13666 for bacterial spot resistance evaluated in each environment (Table 2). The heritability estimates of disease data were low to moderate in the NC 13666 population that ranging from 0.2 to 0.6 (Table 2). Significant correlations (*p<0*.*0001*) were observed between the two locations’ disease data for an individual week and AUDPC (Fig 1C and S1 Table). The PCA bi-plot also showed the possible coalition and a high percent of phenotypic variability was observed between the data sets of bacterial spot (BS) resistance in different environments and locations (Fig 1D). The dimension of the first PC (Dim1) broadly outlined and explained 67.9% of the phenotypic variability for BS resistance at MHCREC-2017 (Fig 1D). The dimension of the second PC (Dim2) also distinguished the 32.1% phenotypic variability for BS resistance (PRS-2017) at opposite angles of the PCA bi-plot (Fig 1D).

**Fig 2:**
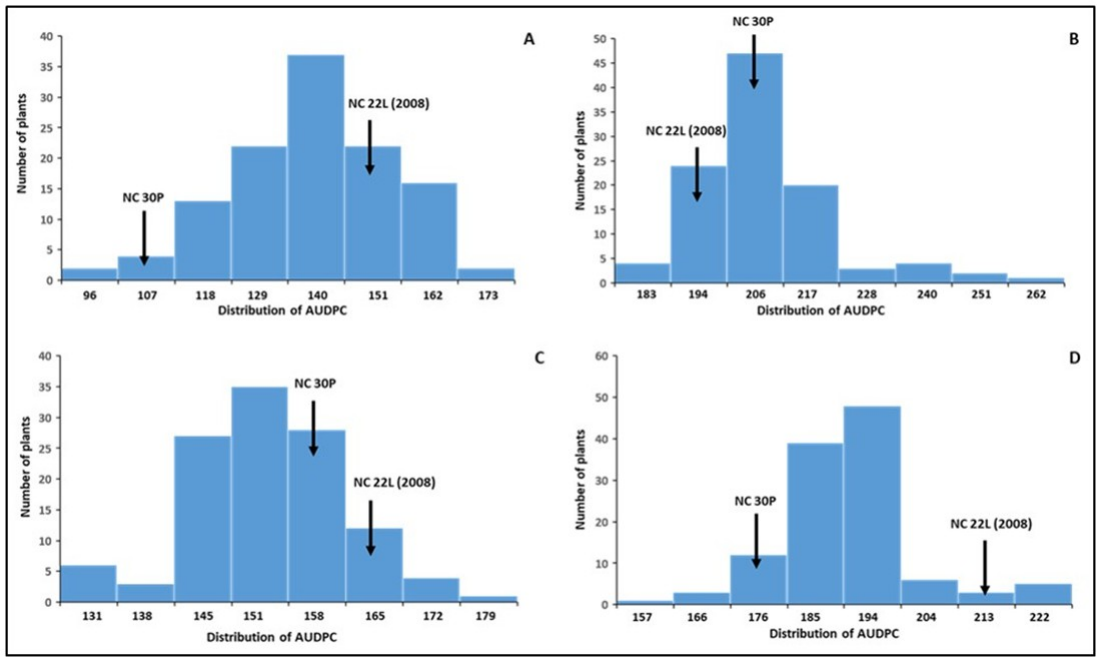
Distribution of Area Under Disease Progress Curve (AUDPC) values for bacterial spot disease severity among NC10204 RIL population across two locations (Mountain Horticultural Crops Research & Extension Center at Mills River- MHCREC, and Piedmont Research Station at Salisbury- PRS) and years. **A**) MHCREC-2016, **B**) PRS-2016, **C**) MHCREC-2017, and **D**) PRS-2017. Arrows indicate the AUDPC values for the parents.

**Fig 3:**
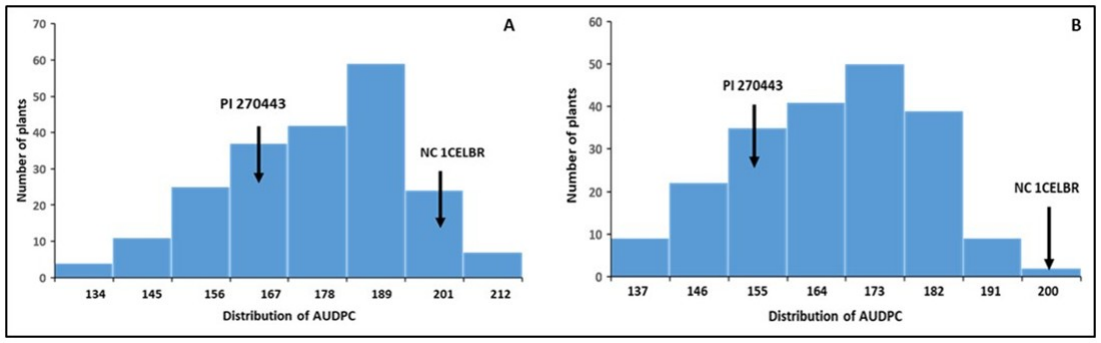
Distribution of AUDPC for bacterial spot disease severity among NC13666 RIL population across two locations. **A**) MHCREC, and **B**) PRS in 2017. Arrows indicate the AUDPC values for the parents.

Although either parental lines of NC 10204 have shown partial resistance in field experiments, we observed a similar level, or more disease, in NC 22L-1(2008) compared to NC 30P (Fig 2). We observed transgressive segregants with a higher and lower level of bacterial spot disease resistance compared to parental lines resulting in a continuous distribution in both populations (Figs 2 and 3). This indicates that both parents are contributing to the favorable alleles for BS resistance and multiple genes are involved in the disease resistance.

### Genetic linkage map

**Fig 4:**
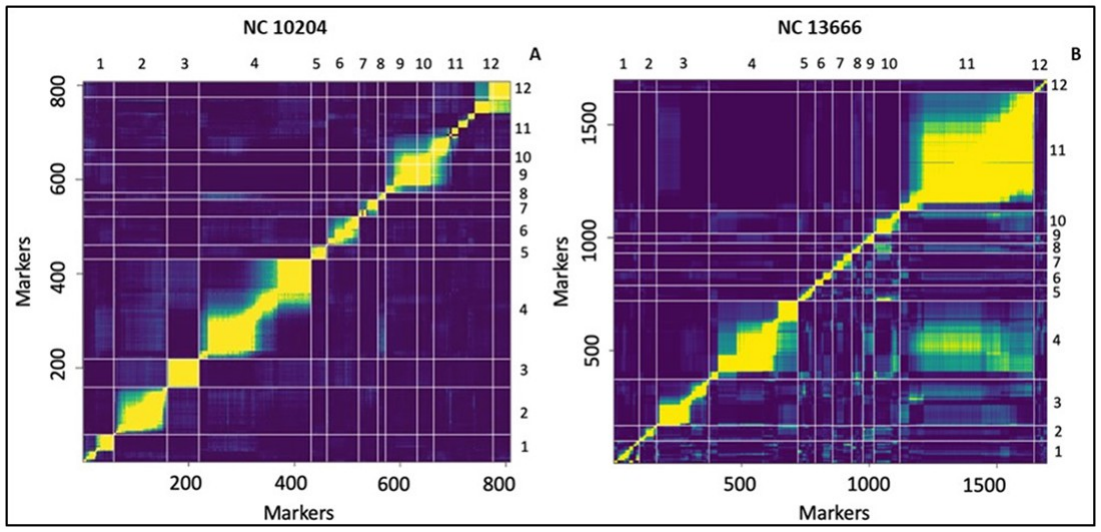
A heat map of a constructed genetic linkage map of A) NC 10204 and B) 13666. The X-axis denotes the arranged markers and chromosome numbers. The Y-axis displays the recombination frequency and LOD scores for all pairs of markers.

The linkage map of NC 10204 has been previously published (Table 3 and S1 Fig) [37]. The NC 13666 linkage map spanned a total of 1395 cM in the genetic distance with an average interval of 1.2 cM among 1699 SNP polymorphic markers (Table 3; S2 Fig and S2 Table). Chromosome 1 had the longest length. Chromosome 10 had the shortest length in the linkage map. The highest number of markers was observed in chromosome 11 (526) in the NC 13666 linkage map (Table 3; S2 Fig and S2 Table). Moreover, a heat map of the mapped markers of NC 10204 and NC 13666 was also constructed using pair-wise recombination values and LOD scores between NC 10204 and NC 13666 genotyped markers (Fig 4). The heat map indicates steady heat across the transverse line within the chromosomes, implying that the maps were constructed accurately (Fig 4).

**Table 3:** Summary of the linkage maps of NC 10204 and NC13666 showing 12 chromosomes along with the number of markers per chromosome and the length of each chromosome.

Chromosome	NC 10204 ^a^		NC 13666	
	Markers	Length (cM)	Markers	Length (cM)
1	58	98.6	99	373.5
2	101	77.4	68	97.4
3	60	52.1	205	92.2
4	212	85.3	348	101.5
5	30	42.6	68	93.1
6	60	66.8	68	167.8
7	36	39.6	75	68.1
8	15	28.8	44	74.4
9	138	94.1	43	102.7
10	31	72.1	101	53.5
11	112	30.1	526	78
12	33	51.9	54	92.7
**Total**	**886**	**739.4**	**1699**	**1394.9**

^a^The map has previously reported in our study (Adhikari et al., 2020)

### QTL analysis of NC 10204 population

**Fig 5:**
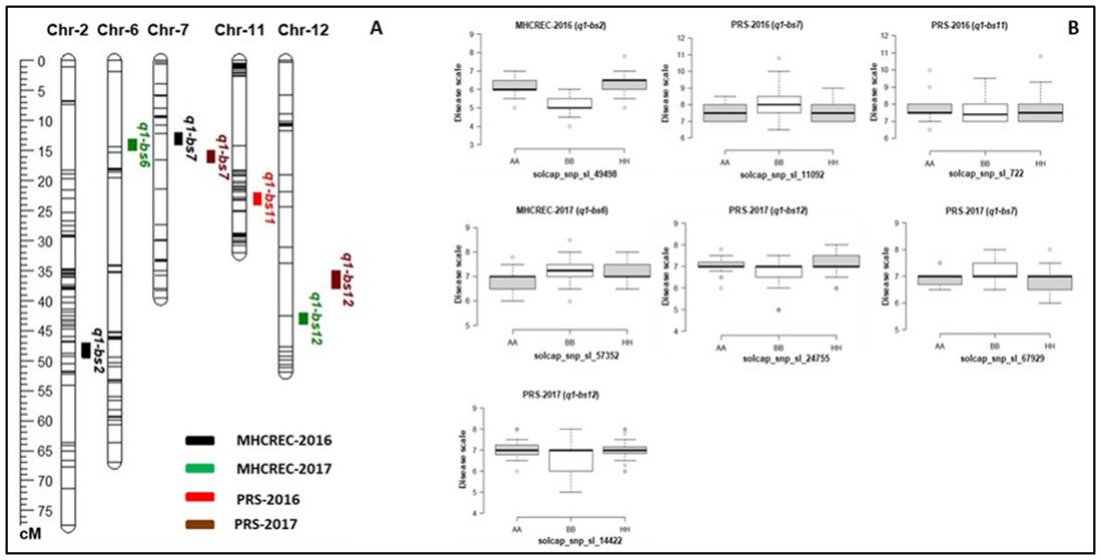
QTL analysis for bacterial spot resistance in mapping population NC10204. **A)** Genetic linkage map showing the locations of BS-resistant QTLs for race T4 with the genetic distance shown in centimorgans (cM) for the NC 10204 population evaluated during 2016–2017 in two locations. **B)** Box plots of resistance level regulated by linked markers to QTLs in NC 10204. Genotypes were grouped based on the associated SNP markers. AA: NC 30P, BB: NC 22L-1(2008), HH: heterozygous.

We identified QTLs for bacterial spot race T4 resistance using 192 RILs and the SNP-based linkage map in two environments during the two years (Fig 5A and Table 4). In total, 7 QTLs, including major and minor effects, common for both environments and those specific to environments were identified across the genome, explaining phenotypic variation (R^2^) ranging from 2.5 to 21.3% (Fig 5A and Table 4). Four QTLs, including on chromosome 7 (*q1-bs7*), and 12 (*q1-bs12*) were detected in at least two environments in NC 10204 population based on composite interval mapping (Fig 5A and Table 4). The QTL on chromosome 6 (*q1-bs6*) with a LOD value of 3.8 (Fig 4) explained the highest percentage of phenotypic variance (up to 21.3%) among the detected QTL, followed by the QTLs on chromosome 12 (LOD = 3.1) that explained up to 7.7 to 8.2% of the total phenotypic variance. The QTL regions on chromosomes 7 and 12 were detected at slightly different positions within (13.1 to 16.6 cM and 36.2 to 44.5 cM) regions in two environments in the genetic map, and the positions of the closest markers associated with the QTL were within ~5Mb and 6Mb in the physical map of tomato, respectively. The QTLs on chromosome 2 (*q1-bs2*) (LOD = 3.2) and 11 (LOD = 3.2) explained up to 2.9% and 2.5 of the phenotypic variance respectively in NC 10204 (Table 4). The box plots of flanking markers revealed that both parents contribute to the BS resistance in the mapping population NC 10204. For the QTLs, *q1-bs2*, *q1-bs6*, *q1-bs11*, *and q1-bs12* alleles from NC 22L-1(2008) increased the level of resistance whereas, QTLs *q1-bs6* and *q1-bs7* had favorable alleles from NC 30P for BS disease resistance (Fig 5B). These results indicated that both parents contributed to the favorable alleles for the bacterial spot race T4 resistance.

**Table 4:** QTL associated with bacterial spot disease resistance against *Xanthomonas perforans* race T4 in an intra-specific mapping population NC10204 of tomato derived from NC 30P x NC 22L-1(2008) in different environments.

Trait	Chromosome	Flanking markers	Position (cM)	Position (Mb)	LOD	R^2^	Additive
MECREC-2016	2	solcap_snp_sl_49498 & solcap_snp_sl_49576	48.7	39–45	3.2	2.9	0.70
PRS-2016	7	solcap_snp_sl_11092 & solcap_snp_sl_67929	13.1	4–9.5	4.4	2.5	-0.38
PRS-2016	11	solcap_snp_sl_722 & solcap_snp_sl_9560	23.1	39–46.5	3.2	2.5	0.09
MECREC-2017	6	solcap_snp_sl_57352 & solcap_snp_sl_57574	14.3	42–45	3.8	21.3	-0.20
MECREC-2017	12	solcap_snp_sl_24755 & solcap_snp_sl_31628	44.5	58–64	3.1	8.2	0.27
PRS-2017	7	solcap_snp_sl_67929 & solcap_snp_sl_53393	16.6	3.8–9	6.0	3.0	-0.14
PRS-2017	12	solcap_snp_sl_14422 & solcap_snp_sl_31628	36.2	60–64	3.1	7.7	-0.20

### QTL analysis of NC 13666 population

**Fig 6:**
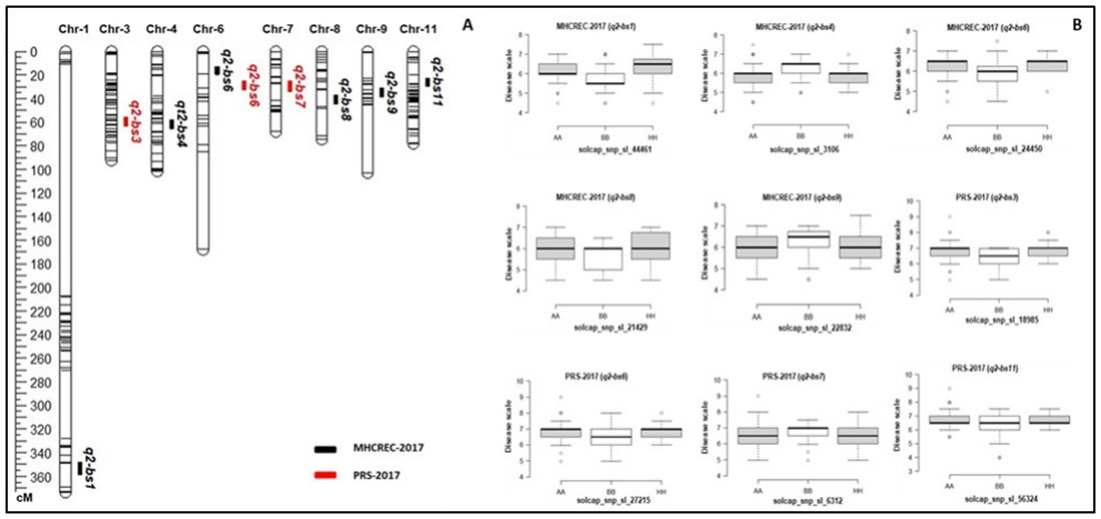
QTL analysis for bacterial spot resistance in mapping population NC 13666. **A)** Genetic linkage map showing the locations of BS-resistant QTLs for race T4 with the genetic distance shown in centimorgans (cM) for the NC 13666 population evaluated during 2017 in two locations. **B)** Box plots of resistance level regulated by linked markers to QTLs in NC 13666. Genotypes were grouped based on the associated SNP markers. AA: NC 1CELBR, BB: NC PI 270443, HH: heterozygous.

The QTL analysis was performed in an additional independent RIL population NC 13666 which had resistance from different parental line. The disease data were collected for only one year in two different environments for this population. In total, 9 QTLs, including major and minor effects, common for both environments and those specific to environments were identified across the genome, explaining phenotypic variation (R^2^) ranging from 4.6 to 23.4% (Fig 6A and Table 5). The QTLs were detected on chromosomes 1, 3, 4, 6, 7, 8, 9, and 11 with LOD ranging from 2.7 to 10.1 (Fig 6A and Table 5). Two QTLs on chromosome 6 were detected in both environments (Fig 6A and Table 5). Although the genetic position of common QTLs differs, the flanking markers share the genomic position (Fig 6A and Table 5). The box plots of flanking markers revealed that both parents contribute to the BS resistance in the mapping population NC 13666. For the QTLs, *q2-bs1*, *q2-bs3*, *q2-bs6*, *q2-bs8*, and *q2-bs11* alleles from PI 270443 increased the level of resistance whereas, QTLs *q2-bs4* and *q2-bs7*, *and q2-bs9* had favorable alleles from NC 1CELBR for BS disease resistance (Fig 6B). These results indicated that both parents contributed to the favorable alleles for the bacterial spot race T4 resistance.

**Table 5:** QTL associated with bacterial spot disease resistance against *Xanthomonas perforans* race T4 in an inter-specific mapping population NC 13666 of tomato derived from NC 1CELBR x PI 270443 in different environments.

Trait	Chromosome	Flanking markers	Position (cM)	Position (Mb)	LOD	R^2^	Additive
MHCREC-17	1	solcap_snp_sl_44461 & solcap_snp_sl_43632	358.6	78–82	3.6	8.3	0.19
MHCREC-17	4	solcap_snp_sl_3106 & solcap_snp_sl_2180	59.3	55–56	5.9	9.0	-0.20
MHCREC-17	6	solcap_snp_sl_24450 & solcap_snp_sl_57594	13.7	32–42.5	10.1	23.4	0.31
MHCREC-17	8	solcap_snp_sl_21429 & solcap_snp_sl_50211	38.4	57–61	2.7	5.6	-0.16
MHCREC-17	9	solcap_snp_sl_22832 & solcap_snp_sl_58001	32.8	4.5–8	3.4	5.0	-0.15
PRS-17	3	solcap_snp_sl_18985 & solcap_snp_sl_30631	58.2	48.3–51.7	7.3	17.8	0.22
PRS-17	6	solcap_snp_sl_27215 & solcap_snp_sl_17019	28.2	42–50	3.0	6.2	0.15
PRS-17	7	solcap_snp_sl_6312 & solcap_snp_sl_6313	26.4	9–17	2.7	4.6	-0.13
PRS-17	11	solcap_snp_sl_56324 & solcap_snp_sl_5970	24.1	46.5–52	3.9	7.2	0.17

### Comparison of QTLs from NC 10204 and NC 13666 populations

We detected common QTLs in both mapping populations. The QTLs on chromosome 7 were detected in the NC 10204 population in PRS-2016 and PRS-2017 (Figs 5A and 6A) and in the NC 13666 population in PRS-2017. These QTL segments shared ~13Mb (4 to 17 Mb) genomic positions on chromosome 7 (Tables 4 and 5). The QTL on chromosome 6 was detected in both locations in NC 13666, and in NC 10204 in MECREC-2017. These QTL segments shared ~18Mb (32 to 50 Mb) genomic positions on chromosome 6 (Tables 4 and 5). The QTL on chromosome 11 was detected in both populations in PRS-2016 and PRS-2017. These QTLs shared ~13Mb (39 to 52 Mb) genomic positions on chromosome 11 (Tables 4 and 5). These common QTLs in two independent mapping populations can serve as reliable loci to breed BS resistance in elite tomato varieties.

## Discussion

Bacterial spot disease has been a significant problem in tomato production regions for more than 60 years. However, neither chemical control nor breeding for host resistance has achieved durable success. New races of the pathogen have evolved over time, and the distribution of the pathogen species has shifted many times. Currently, *X*. *perforans* race T4 is a major problem in tomato production regions in Southeast US including NC [12, 20]. Therefore, we sought to identify QTL and eventually develop molecular markers associated with the bacterial spot race T4 resistance in an intra-specific population, so that they can be utilized in the breeding program without the concern of linkage drag. We also evaluated another inter-specific population for BS resistance to support our results from the intra-specific population. We identified four QTLs on chromosomes 6, 7, and 11 associated with BS race T4 resistance in two independent RIL populations. Both parents of each population contributed to resistance alleles in our study. Previously, resistance alleles for early blight resistance also came from both parents [46, 47].

The phenotypic data for disease severity was collected in the same two locations PRS and MHCREC for both NC 10204 and NC 13666. We observed high disease pressure in PRS compared to MHCREC. This might be due to different environmental conditions in the two locations. MHCREC is cooler than the PRS site, and PRS had favorable weather conditions for bacterial spot disease progress, i.e. warm, humid conditions and high rainfall with the optimum temperature of 24°C to 30°C [48, 49].

In two populations, we detected QTL for BS resistance in all chromosomes except chromosomes 2, 5, and 12. The alleles associated with disease resistance showing the highest phenotypic variations were from NC 30P in the NC 10204 population, and from PI 270443 in the NC 13666 population. This suggests NC 30P could be used as a source of bacterial spot resistance in the breeding program.

Previously, the resistance gene *RxopJ4* effective against race T4 race was mapped to a 190- kb segment on the long arm of chromosome 6 between markers J350 and J352 in *S*. *pennellii* LA 716 [21]. The genomic position of J350 is 35036517 to 35036818 and J352 is 35241322 to 35241736 [21]. In our study, the q1-bs6 and q2-bs6 were located on chromosome 6 in the genomic region of 42 Mb to 50Mb in NC 10204 and NC 13666 mapping populations, which are close to the location of *RxopJ4*, although the parental sources of this resistance were different. Therefore, the QTL detected in our study on chromosome 6 could be allelic to the *RxopJ4* gene, but further investigation is required to verify it.

There is also a possibility that the QTLs on chromosome 6 are associated with the plant growth habit. Since NC 10204 was segregating for plant growth habits, individual plants with indeterminate growth habits might appear resistant compared to determinate growth habits. A study has reported that the bacterial spot disease resistance against race T1 was affected by the plant growth habit [50]. The self-pruning (*sp*) gene is also located on chromosome 6 at the position of ~46 Mb according to the gene information available on Sol Genomics Network (SGN) [39], which is close to the position of the QTL for bacterial spot disease resistance in our study (~42Mb to ~50Mb). This necessitates confirming further if the QTL on chromosome 6 detected in this study is segregating for the bacterial spot disease resistance, the growth habit, or both. Since *q1-bs6* explained the highest percentage of phenotypic variance in NC 10204, this QTL might be useful in the tomato breeding program if further studied, as there will be minimal linkage drag, unlike in LA 716.

The *q2-bs1* explained the phenotypic variance (up to 8.3%) in NC 13666. In a previous study, a minor QTL was detected on chromosome 1 for race T4 at TOM 202 marker flanked by *LEVCOH11* and *LEVCOH12* markers in PI 114490 derived IBC population, but could not be confirmed through a selective genotypic approach [14]. A preliminary study identified a locus in chromosome 1 against race T4 that was derived from a susceptible parent [13]. As we detected the QTL on chromosome 1, this represents a novel QTL against race T4. However, the QTL was detected on different positions of chromosome 1 in different environments. Therefore, the QTL detected on chromosome 1 in different environments might represent two different QTLs, or the environment is heavily influencing the QTL identity.

Another QTL was detected on chromosome 4 in NC 13666 population explaining up to 9% of phenotypic variance. No other QTL has been reported on chromosome 4 against race T4 of bacterial spot disease so far in tomato genotypes. The *q2-bs4* was in the genomic region of 55 Mb to 59 Mb in NC 13666 in the physical map. The alleles associated with the disease resistance on chromosome 4 were derived from NC 1CELBR in NC 13666 population. The QTL detected on chromosome 4 in our study represents a novel QTL and provides useful information for the breeding of tomatoes against bacterial spot disease race T4. The minor QTL on chromosome 2 in NC10204 was also detected. However, [13] identified a QTL with a strong effect on chromosome 2 against race T4 in a PI 114490-derived population in a preliminary study.

Although *q1-bs11* and *q2-bs11* were detected in NC 10204 and NC 13666 populations in our study, [14] identified QTL on chromosome 11 (explained 29.4% phenotyping variance) and 3 (explained 4.8% phenotypic variance) against race T4 in PI 114490-derived inbred backcross population and confirmed through selective genotyping in three populations derived from three breeding lines Fla. 8517, Fla. 8326, and Fla. 8517. Another preliminary study identified seven robust QTLs, one in chromosome 3, and two in each of chromosomes 2, 10, and 11; three weak QTLs on chromosomes 8, 9, and 12 from PI 114490, whereas QTLs on chromosomes 1, 7, and 9 were from the susceptible parent [13]. In our study the QTL on chromosome 12 was also detected, contributing to 7.7% of phenotypic variance. In a recent study, a research group introgressed the 3 segments of QTLs 11 (*QTL-11A*, *QTL11-B*, and *QTL-11C*) along with the combination of *QTL-11A* with *Rx-4/Xv3* [6]. According to their results, *QTL-11A* and *QTL-11C* combined with *Rx-4/Xv3* displayed maximum resistance against multiple races [6]. These results showed the introgression of various QTL segments can provide resistance against multiple species and races.

In summary, we identified major QTLs associated with bacterial spot disease resistance on chromosomes 6, 7, and, 11 in both intra-specific and inter-specific mapping populations. The donor of the resistance associated with the genomic regions on chromosome 6 in NC 10204 that explained the highest percentage of phenotypic variance was NC 30P, a released breeding line with superior horticultural traits. Therefore, NC 30P would be a valuable resource for tomato breeding programs to further work on bacterial spot disease resistance breeding. NC 30P can be utilized successfully as a parent without much concern about the linkage drag as observed in the case of LA 716. NC 30P also contributed to the resistance associated with the genomic region on chromosome 7. Although the resistance associated with the region on chromosomes 2, 11, and 12 was obtained from NC 22l-1(2008), this can be useful in stacking of resistance QTLs. Furthermore, the SNP markers associated with BS race T4 resistance can be used for marker-assisted selection.

## Supporting information

S1 Fig Genetic map of the NC 10204 mapping population. (TIF)

S2 Fig Genetic map of the NC 10204 mapping population. (TIF)

S1 Table The correlation coefficient of the disease response between generations and locations. (DOCX)

S2 Table Geno and mapping data. (XLSX)
