# Supplementary figures and images for: Identification of quantitative trait loci associated with bacterial spot race T4 resistance in intra-specific populations of tomato (Solanum lycopersicum L.)
Source: PLoS One. 2023 Dec 11;18(12):e0295551. doi: 10.1371/journal.pone.0295551 (PMC10712892; doi:10.1371/journal.pone.0295551)

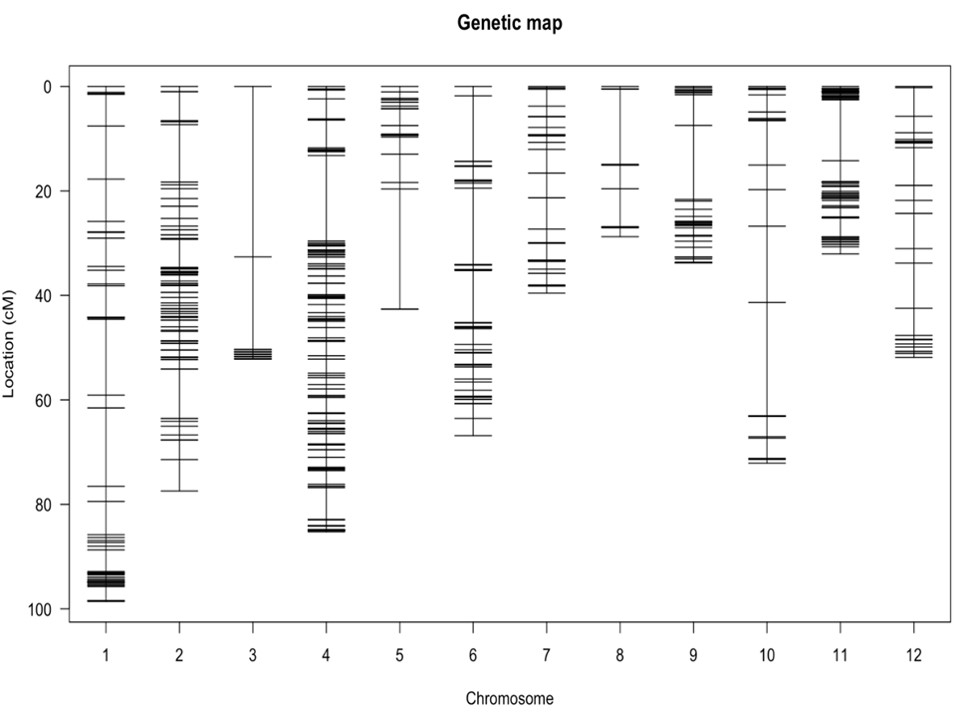

Supplement: S1 Fig — (TIF) [file pone.0295551.s001.tif]

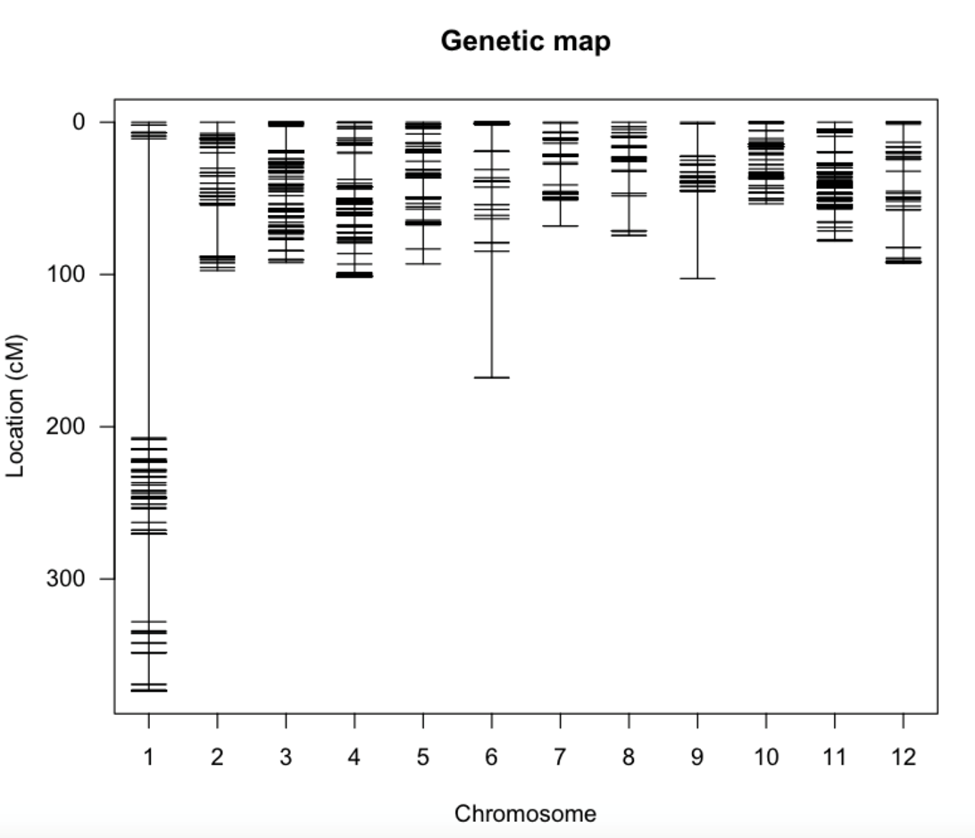

Supplement: S2 Fig — (TIF) [file pone.0295551.s002.tif]
