# Supplementary material for: Identification of quantitative trait loci associated with bacterial spot race T4 resistance in intra-specific populations of tomato (Solanum lycopersicum L.)
Source: PLoS One. 2023 Dec 11;18(12):e0295551. doi: 10.1371/journal.pone.0295551 (PMC10712892; doi:10.1371/journal.pone.0295551)
Supplement: S1 Table — (DOCX) [file pone.0295551.s003.docx]

**Supplementary table**

**Table S1** The correlation coefficients of the disease response between two generations and two locations in the intra-specific population NC 10204. SCORE 1-5 represent disease severity measured according to [Horsfall and Barratt (1945](#_ENREF_13)) scale for week 1-5 respectively.

| **Variables** | **Correlation (r) between years2016 and 2017** | | **Correlation (r) between locations MHCREC and PRS** | |
| --- | --- | --- | --- | --- |
|  | **Location=MHCREC** | **Location=PRS** | **Year=2016** | **Year=2017** |
| **AUDPC** | 0.3**^a^ | 0.21* | ns | ns |
| **SCORE1** | ns^b^ | ns | ns | ns |
| **SCORE2** | ns | ns | ns | 0.3** |
| **SCORE3** | 0.3** | 0.3** | ns | ns |
| **SCORE4** | 0.2* | ns | 0.2* | ns |
| **SCORE5** | 0.3** | 0.4**** | 0.4**** | ns |

**Note**: ^a^ ‘****’ denotes p - value < 0.0001, ‘**’ denotes p - value < 0.01, ‘*’ denotes p - value < 0.05, and ^b^ ns represents non-significant correlations.
